# Supplementary figures and images for: Systematic identification of molecular mediators of interspecies sensing in a community of two frequently coinfecting bacterial pathogens
Source: PLoS Biol. 2022 Jun 21;20(6):e3001679. doi: 10.1371/journal.pbio.3001679 (PMC9249247; doi:10.1371/journal.pbio.3001679)

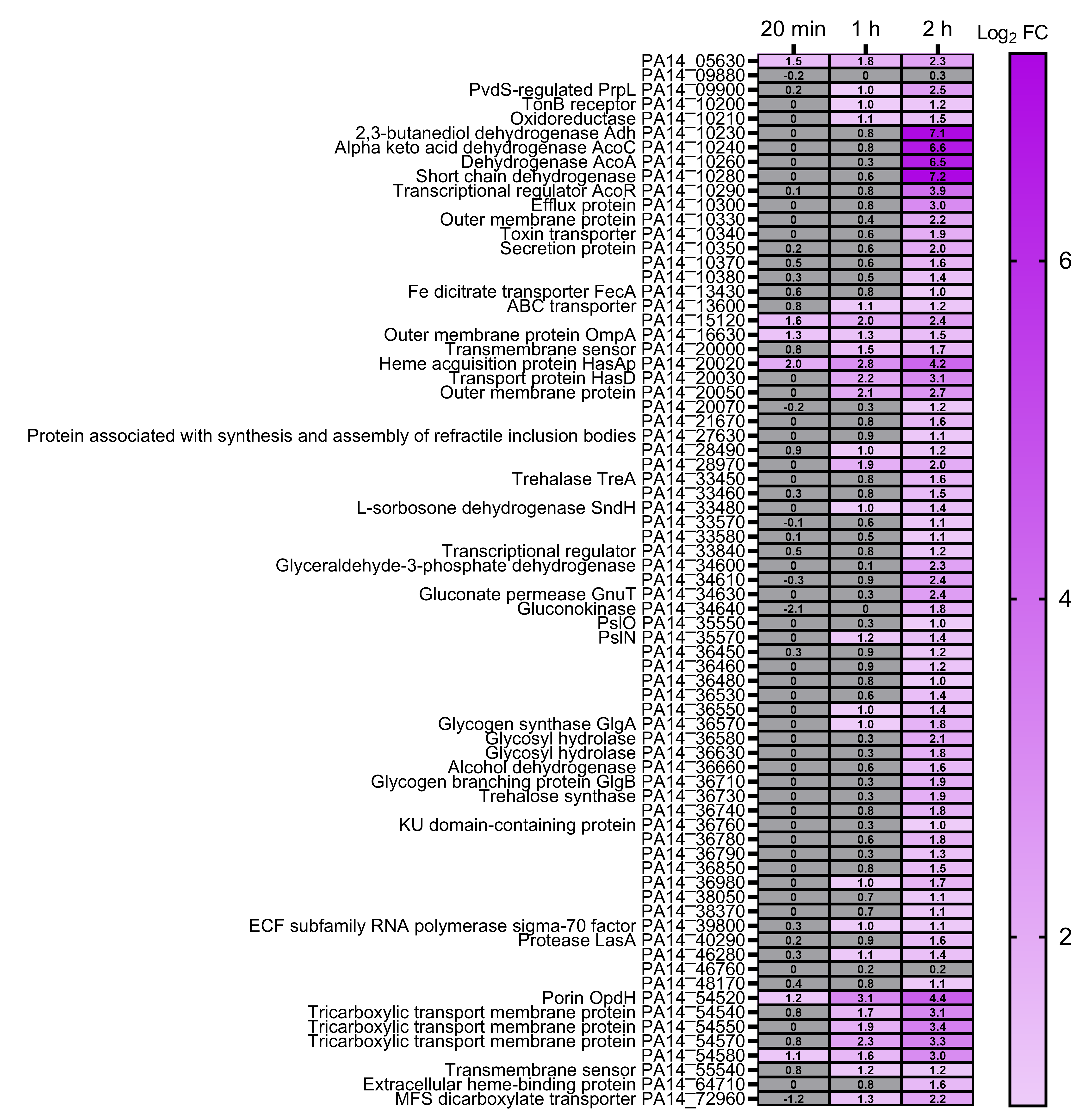

Supplement: S1 Fig — The log2 fold changes for all up-regulated genes with increasing fold change over time from 20 minutes to 1 hour to 2 hours are shown. Gray, log2 fold change < 1. The data underlying this figure can be found in Tables A and B in S1 File. (TIF) [file pbio.3001679.s001.tif]

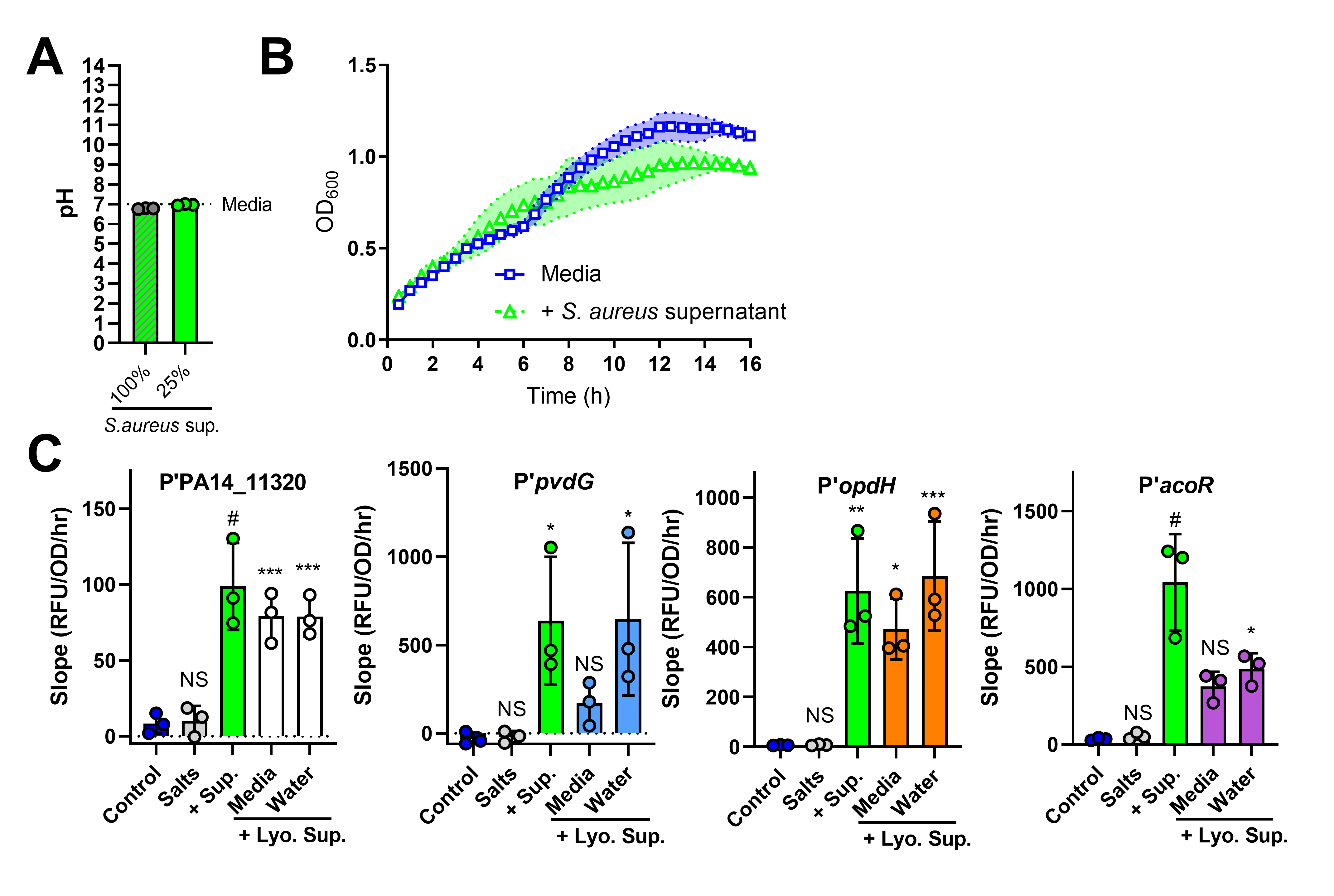

Supplement: S2 Fig — (A) The pH of 100% S. aureus supernatant and 25% (v/v) S. aureus supernatant in medium was measured. (B) Growth of P. aeruginosa was measured after exposure to 25% S. aureus supernatant or media control during the plate reader assay. (C) RFUs of mScarlet normalized to OD600 over time after exposure to media control, the salts from the media, S. aureus supernatant, or lyophilized S. aureus supernatant resuspended in either media or water, in the indicated promoter-reporter strains. Slope calculated from 1.5 to 5 hours (promoters of PA14_11320, opdH, and acoR) or 1 to 4 hours (promoter of pvdG). Datasets were analyzed by 1-way ANOVA with Dunnett test for multiple comparisons to the media control. Data shown for all panels are the means of 3 independent biological replicates, including 3 separate supernatant collections in A. Shaded regions and error bars denote the SD. *, p < 0.05; **, p < 0.01; ***, p < 0.001; #, p < 0.0001. The data underlying all panels can be found in S1 Table. RFU, relative fluorescence unit; SD, standard deviation. (TIF) [file pbio.3001679.s002.tif]

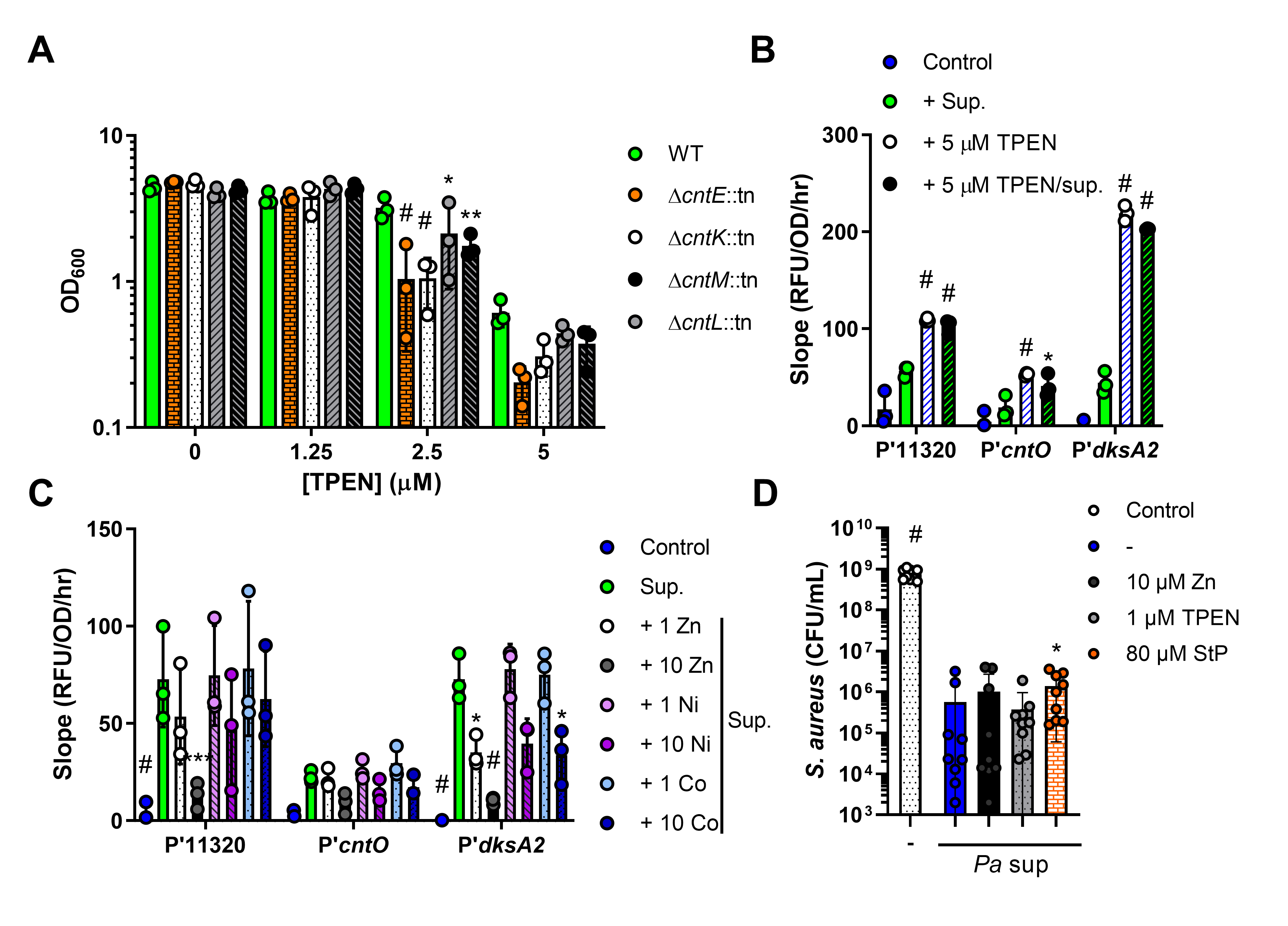

Supplement: S3 Fig — (A) WT S. aureus and NTML transposon mutants were grown in media with or without the addition of the zinc chelator TPEN at the concentrations indicated. Growth was measured at OD600 after 24 hours. Datasets were analyzed by a 2-way ANOVA with Dunnett test for multiple comparisons to the respective WT value. (B, C) RFU of mScarlet normalized to OD600 over time after exposure to media control or S. aureus supernatant and/or addition of the indicated concentrations of (B) TPEN or (C) zinc, nickel, and cobalt in the indicated P. aeruginosa promoter-reporter strains (promoters of PA14_11320, cntO, or dksA2). Datasets were analyzed by a 2-way ANOVA with Tukey test for multiple comparisons. Statistics shown represent tests comparing (B) the respective conditions/promoters with and without TPEN and (C) all treatments to the respective supernatant addition. (D) S. aureus cultures were grown in culture with 50% medium salts (Control) or cell-free supernatants of P. aeruginosa grown in the presence of the indicated additives. Colony-forming units per milliliter of S. aureus were calculated after 16 hours of growth. Datasets were analyzed by 1-way ANOVA with Dunnett test for multiple comparisons to the S. aureus grown in supernatant from P. aeruginosa grown in media without additions. Data shown for all panels are the means of 3 independent biological replicates, including 3 separate supernatant collections in D. The error bars denote the SD. *, p < 0.05; **, p < 0.01; ***, p < 0.001; #, p < 0.0001. The data underlying all panels can be found in S1 Table. NTML, Nebraska Transposon Mutant Library; RFU, relative fluorescence unit; SD, standard deviation; StP, staphylopine; WT, wild type. (TIF) [file pbio.3001679.s003.tif]

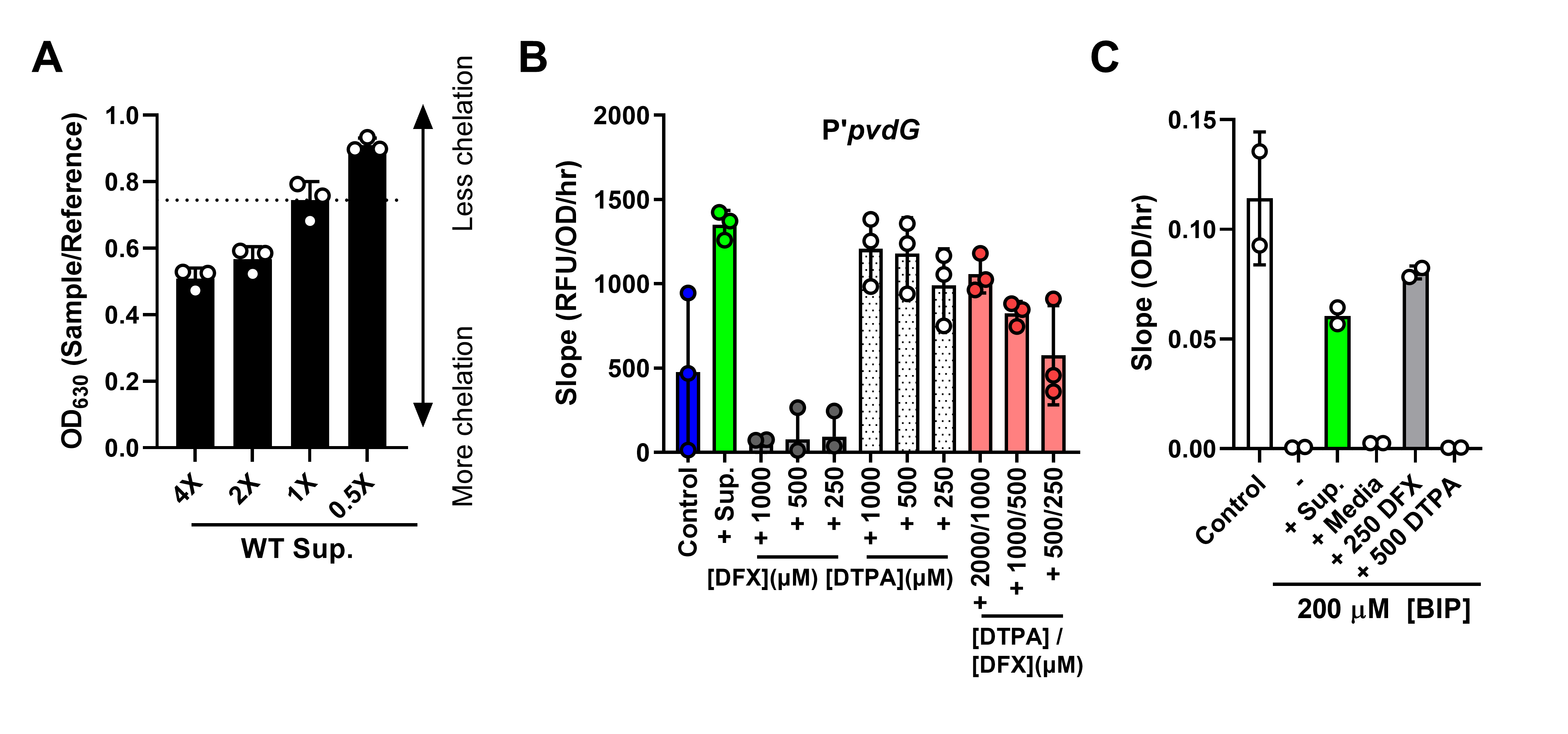

Supplement: S4 Fig — (A) Chelation by different amounts of S. aureus supernatant was measured by CAS assay to construct a standard curve to calculate relative chelation. (B) RFU of mScarlet expressed from the promoter of pvdG normalized to OD600 over time after exposure to media control, S. aureus supernatant, or the indicated concentrations of the iron chelators DFX and DTPA. Data shown from 3 independent replicates. (C) Slope of OD600 over time from 1 to 8 hours of P. aeruginosa ΔpvdJ ΔpchE strain in medium control or medium with 200 μM BIP with the addition of whole WT S. aureus supernatant, media, or the iron chelators DFX or DTPA. Error bars denote the SD. The data underlying all panels can be found in S1 Table. CAS, chromeazurol S; DFX, deferoxamine; DTPA, diethylene triamine penta-acetic acid; RFU, relative fluorescence unit; SD, standard deviation; WT, wild type. (TIF) [file pbio.3001679.s004.tif]

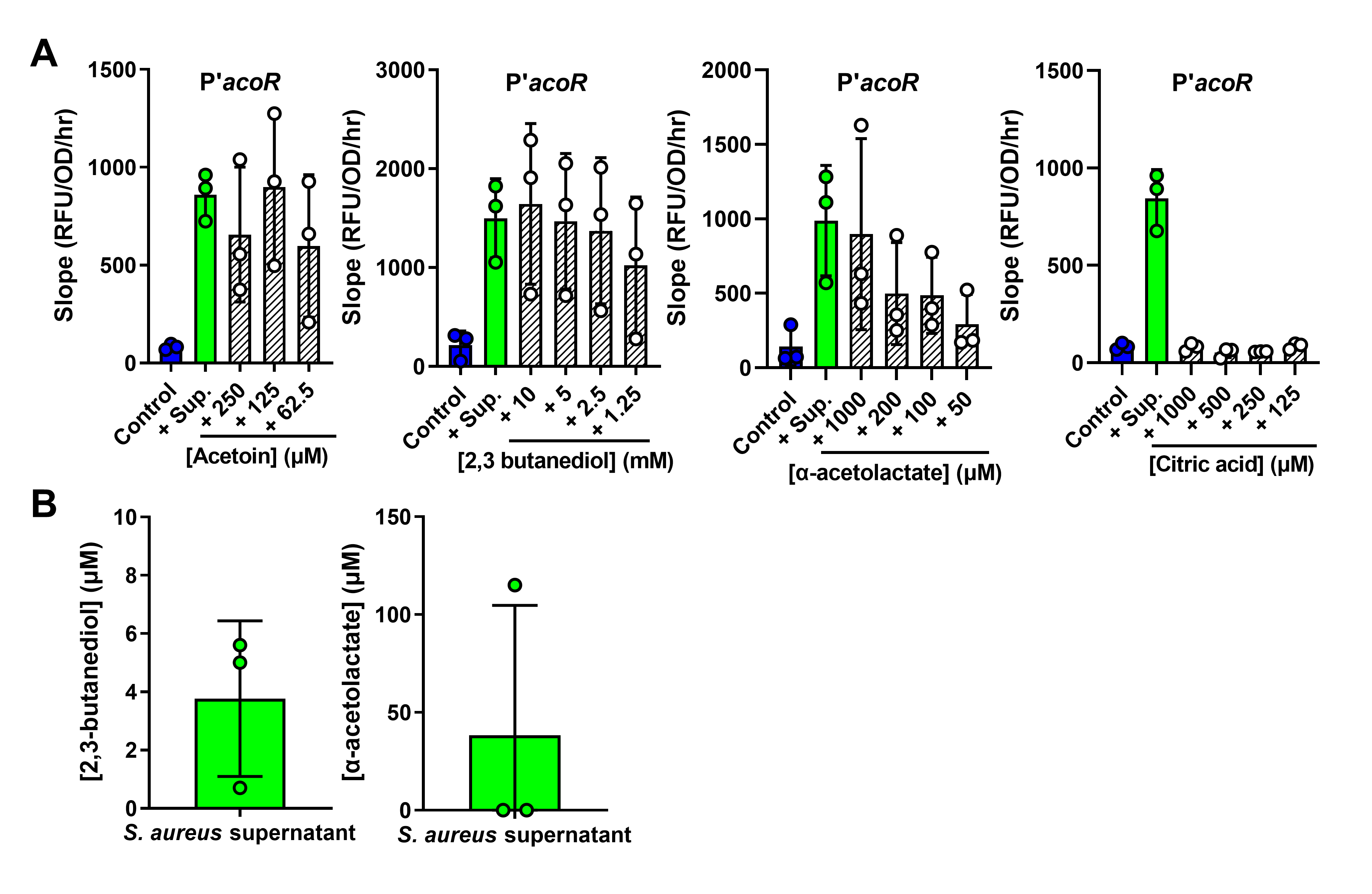

Supplement: S5 Fig — (A) RFU of mScarlet expressed from the acoR promoter normalized to OD600 over time after exposure to media control, S. aureus supernatant, or addition of the indicated concentrations of each metabolite. (B) Quantification of 2,3-butanediol and α-acetolactate in S. aureus supernatant. Data shown for all panels is from 3 independent replicates. Error bars denote the SD. The data underlying all panels can be found in S1 Table. RFU, relative fluorescence unit; SD, standard deviation. (TIF) [file pbio.3001679.s005.tif]

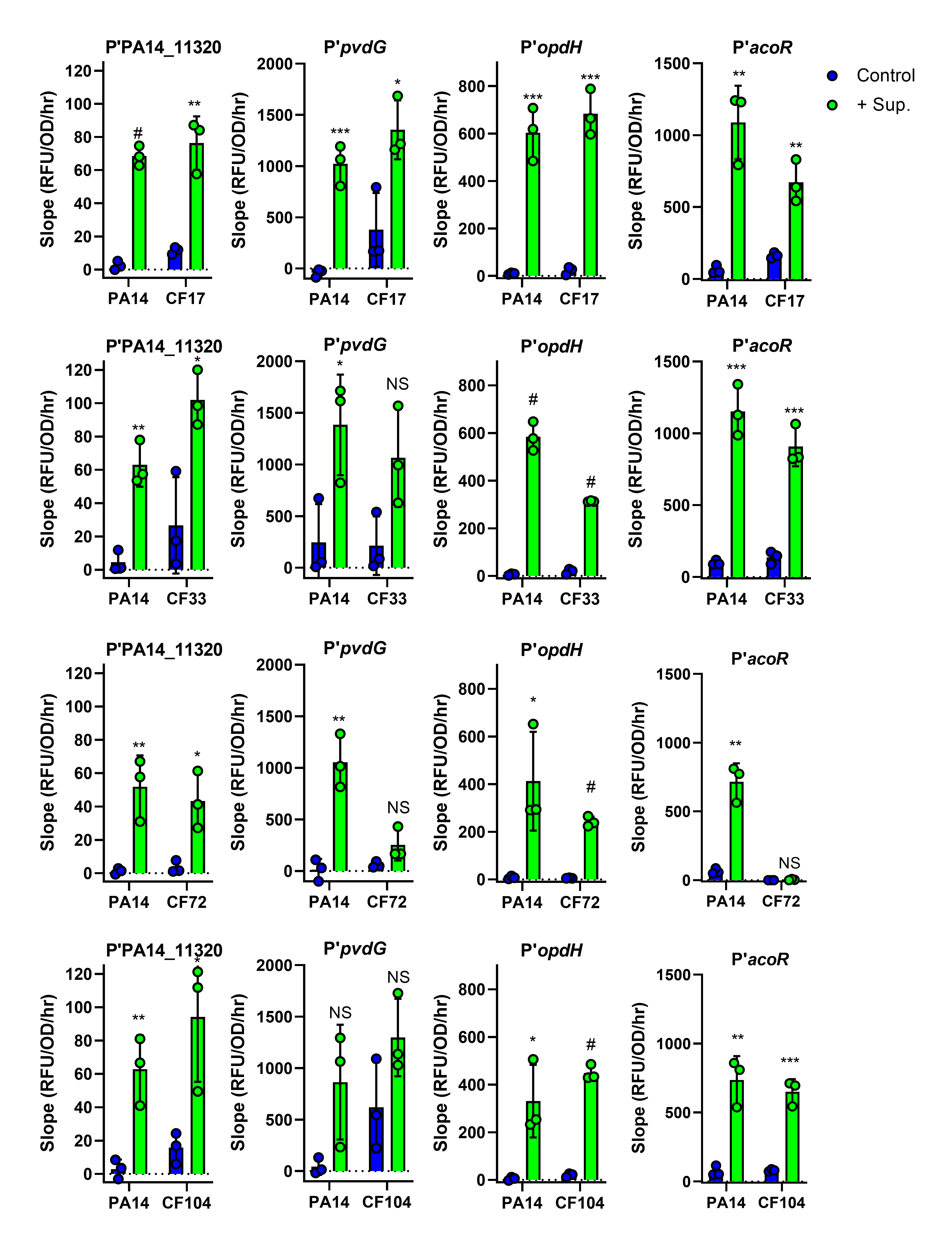

Supplement: S6 Fig — RFU of mScarlet normalized to OD600 over time after exposure to S. aureus supernatant or media control from the indicated promoter-reporter constructs in P. aeruginosa PA14 and clinical isolate strains CF17, CF33, CF72, and CF104. Slope calculated from 1.5 to 5 hours. Data shown for all panels are the means of 3 independent biological replicates. Datasets were analyzed by unpaired t tests to the respective media control. The error bars denote the SD. *, p < 0.05; **, p < 0.01; ***, p < 0.001; #, p < 0.0001. The data underlying all panels can be found in S1 Table. RFU, relative fluorescence unit; SD, standard deviation. (TIF) [file pbio.3001679.s006.tif]

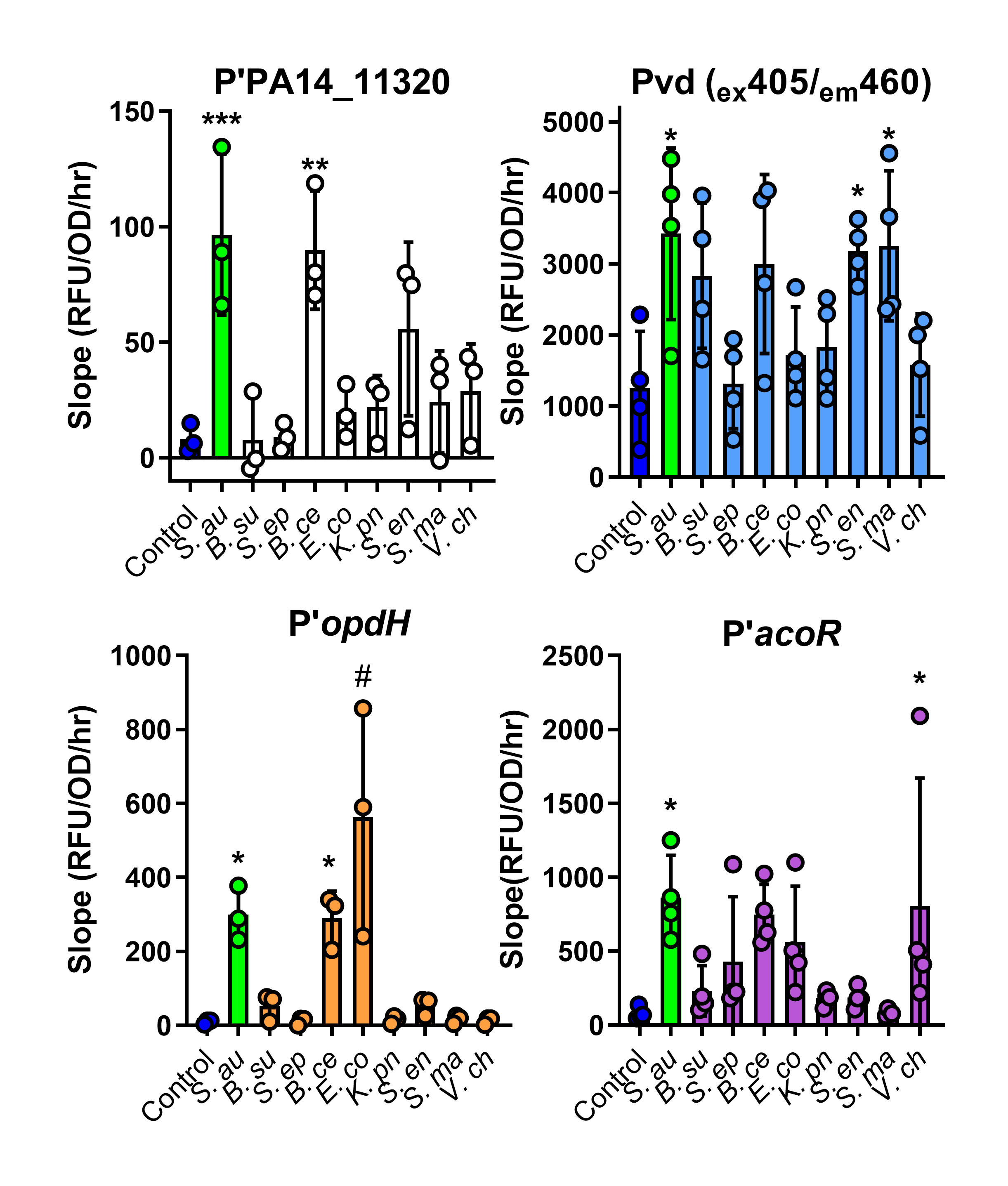

Supplement: S7 Fig — RFU of mScarlet expressed from the indicated promoter or Pvd normalized to OD600 over time (promoter of PA14_11320, opdH, and acoR calculated from 1.5 to 5 hours; Pvd calculated from 1 to 4 hours) after exposure to media control, S. aureus supernatant, or supernatant from the indicated species: Bacillus subtilis, Staphylococcus epidermidis, Burkholderia cenocepacia, Escherichia coli, Klebsiella pneumoniae, Salmonella enterica Typhimurium, Stenotrophomonas maltophilia, and Vibrio cholerae. Data shown from at least 3 independent replicates. Error bars denote the SD. Datasets were analyzed by 1-way ANOVA with Dunnett test for multiple comparisons to the control. *, p < 0.05; **, p < 0.01; ***, p < 0.001; #, p < 0.0001. The data underlying all panels can be found in S1 Table. Pvd, pyoverdine; RFU, relative fluorescence unit; SD, standard deviation. (TIF) [file pbio.3001679.s007.tif]

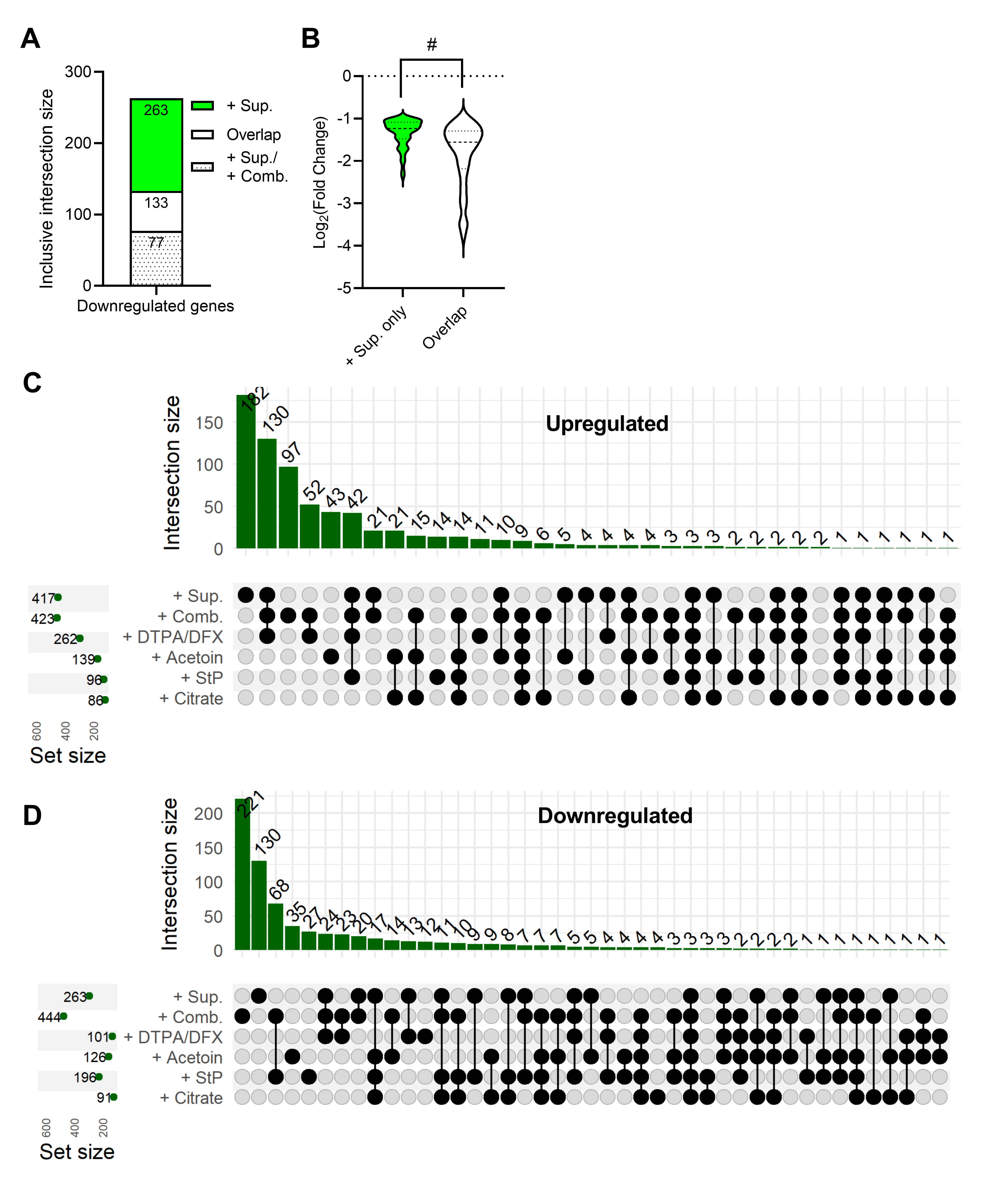

Supplement: S8 Fig — (A) Inclusive intersection of all down-regulated genes after addition of supernatant (+ Sup.) versus at least one other condition (Overlap) or the combination of all molecules (+ Sup. / + Comb.). (B) Log2 fold change of the down-regulated genes that are nonintersecting (+ Sup. only) or intersecting among supernatant and at least one other condition (Overlap). Medians (dashed lines) and first/third quartiles (dotted lines) are shown. Datasets were analyzed by a 2-tailed t test. #, p < 0.0001. (C, D) UpSet plot showing all exclusive intersections of (C) up-regulated or (D) down-regulated genes at 20 minutes and 2 hours after addition of S. aureus supernatant, the indicated products, or the combination of all products (+ Comb.). All intersections are shown. The data underlying panels ACD and B can be found in Table M in S1 File and S1 Table, respectively. (TIF) [file pbio.3001679.s008.tif]
