# Supplementary material for: Systematic identification of molecular mediators of interspecies sensing in a community of two frequently coinfecting bacterial pathogens
Source: PLoS Biol. 2022 Jun 21;20(6):e3001679. doi: 10.1371/journal.pbio.3001679 (PMC9249247; doi:10.1371/journal.pbio.3001679)
Supplement: S2 Table — (PDF) [file pbio.3001679.s011.pdf]

S2 Table. Bacterial strains and plasmids used in this study.

Name	Description[a]	Source
**Strains[b]**		
P. aeruginosa		
PA14	University of California Berkeley Plant Pathology (UCBPP)-PA14	(1)
AK625	Δ*pvdJ* Δ*pchE*	(2)
CF17	AMT0194-10; co-isolated with *S. aureus*; age: 4.91 years	CFF Isolate Core
CF33	AMT0457-07; co-isolated with *S. aureus*; age: 4.28 years	CFF Isolate Core
CF72	AMT0482-03; co-isolated with *S. aureus*; age: 9.06 years	CFF Isolate Core
CF104	AMT0504-19; co-isolated with *S. aureus*; age: 4.70 years	CFF Isolate Core
SB89	PA14 P'*dksA2*-mScarlet (pSB83) unmarked	This study
SB91	PA14 P'11320-mScarlet (pSB85) unmarked	This study
SB124	PA14 P'*opdH*-mScarlet (pSB109) unmarked	This study
SB136	PA14 P'*acoR*-mScarlet (pSB121) unmarked	This study
SB141	PA14 Δ*cntO* P'11320-mScarlet (pSB85) unmarked	This study
SB142	PA14 Δ*cntI* P'11320-mScarlet (pSB85) unmarked	This study
SB143	PA14 Δ*cnt* P'11320-mScarlet (pSB85) unmarked	This study
SB204	PA14 P'*pvdG*-mScarlet (pSB175) unmarked	This study
SB301	CF17 P'11320-mScarlet (pSB85); Gent^r	This study
SB302	CF17 P'*opdH*-mScarlet (pSB109); Gent^r	This study
SB303	CF17 P'*acoR*-mScarlet (pSB121); Gent^r	This study
SB304	CF17 P'*pvdG*-mScarlet (pSB175); Gent^r	This study
SB305	CF33 P'11320-mScarlet (pSB85); Gent^r	This study
SB306	CF33 P'*opdH*-mScarlet (pSB109); Gent^r	This study
SB307	CF33 P'*acoR*-mScarlet (pSB121); Gent^r	This study
SB308	CF33 P'*pvdG*-mScarlet (pSB175); Gent^r	This study
SB309	CF72 P'11320-mScarlet (pSB85); Gent^r	This study
SB310	CF72 P'*opdH*-mScarlet (pSB109); Gent^r	This study
SB311	CF72 P'*acoR*-mScarlet (pSB121); Gent^r	This study
SB312	CF72 P'*pvdG*-mScarlet (pSB175); Gent^r	This study
SB313	CF104 P'11320-mScarlet (pSB85); Gent^r	This study
SB314	CF104 P'*opdH*-mScarlet (pSB109); Gent^r	This study
SB315	CF104 P'*acoR*-mScarlet (pSB121); Gent^r	This study
SB316	CF104 P'*pvdG*-mScarlet (pSB175); Gent^r	This study
S. aureus		
JE2	*Staphylococcus aureus* subsp. *aureus* USA300_FPR3757 (CA-MRSA)-JE2	(3)
CF049	AMT0150-13; age: 8.05 years	CFF Isolate Core
CF061	AMT0150-28; co-isolated with *P. aeruginosa*; age: 15.58 years	CFF Isolate Core
CF085	AMT0458-3; co-isolated with *P. aeruginosa*; age: 9.90 years	CFF Isolate Core
CF089	AMT0461-13; co-isolated with *P. aeruginosa*; age: 12.16 years	CFF Isolate Core

E. coli		
ccdB Survival 2 T1[R]	*E. coli* strain used for maintenance of pDONR plasmid	Invitrogen
DH5α	*E. coli* strain used for cloning	NEB
AK111	MG1655 SB144	(4)
S17-1 λ-pir	*E. coli* strain used for conjugation	(5)
Other species		
SB80	*Staphylococcus epidermidis* (Winslow and Winslow) Evans FDA strain PCI 1200; ATCC 12228	ATCC
SB81	*Salmonella enterica* subsp. *Enterica* (ex Kauffmann and Edwards) Le Minor and Popoff serovar Typhimurium; ATCC 29630	ATCC
SB145	*Bacillus subtilis* PY79	K. Ramamurthi
SB146	*Burkholderia cenocepacia*; ATCC 25608	S. Adhya
SB147	*Klebsiella pneumoniae* subsp. *pneumoniae* KPNIH1	S. Adhya
SB148	*Stenotrophomonas maltophilia* (Hugh) Palleroni and Bradbury K279a; ATCC BAA-2423	ATCC
SB149	*Vibrio cholerae*	S. Adhya
**Plasmids**		
Promoter-reporter		
pSEK109	pLD3208. Shuttle vector with FRT sites and *mScarlet* ORF; Tet^r Gent^r	(6)
pSB83	pSEK109: P'PA14_73020 (*dksA2*)	This study
pSB85	pSEK109: P'PA14_11320	This study
pSB109	pSEK109: P'PA14_54520 (*opdH*)	This study
pSB118	pSEK109: P'PA14_63960 (*cnt*)	This study
pSB121	pSEK109: P'PA14_10290 (*acoR*)	This study
pSB175	pSEK109: P'PA14_33270 (*pvdG*)	This study
Gene deletion		
pDONRPEX 18Gm	Shuttle vector with *attP* sites and *ccdB*; Cm^r Gent^r	(7)
pSB138	pDONRPEX18Gm: ΔPA14_63960 (*cntO*)	This study
pSB139	pDONRPEX18Gm: ΔPA14_63910 (*cntI*)	This study
pSB140	pDONRPEX18Gm: ΔPA14_63960-PA14_63910 (*cnt*)	This study
Remove antibiotic resistance cassette		
pFLP2	Expressing Flp recombinase; Ap^r Car^r	(8)

a Ap^r, ampicillin resistance (*E. coli*); Car^r, carbenicillin resistance (*P. aeruginosa*); Cm^r, chloramphenicol resistance (*E. coli*); Gent^r, gentamicin resistance (*P. aeruginosa*); Tet^r, tetracycline resistance (*E. coli*)

b CFF Isolate Core Samples are annotated as follows: AMT####-## (Patient ID-Isolate number)

SUPPORTING REFERENCES

1. Rahme LG, Stevens EJ, Wolfort SF, Shao J, Tompkins RG, Ausubel FM. 1995. Common virulence factors for bacterial pathogenicity in plants and animals. Science 268:1899-902.

2. Khare A, Tavazoie S. 2015. Multifactorial competition and resistance in a two-species bacterial system. PLoS Genet 11:e1005715.

3. Kennedy AD, Otto M, Braughton KR, Whitney AR, Chen L, Mathema B, Mediavilla JR, Byrne KA, Parkins LD, Tenover FC, Kreiswirth BN, Musser JM, DeLeo FR. 2008. Epidemic community-associated methicillin-resistant *Staphylococcus aureus*: recent clonal expansion and diversification. Proc Natl Acad Sci U S A 105:1327-32.

4. Guyer MS, Reed RR, Steitz JA, Low KB. 1981. Identification of a sex-factor-affinity site in *E. coli* as gamma delta. Cold Spring Harb Symp Quant Biol 45 Pt 1:135-40.

5. de Lorenzo V, Timmis KN. 1994. Analysis and construction of stable phenotypes in gram-negative bacteria with Tn5- and Tn10-derived minitransposons. Methods Enzymol 235:386-405.

6. Wang B, Lin YC, Vasquez-Rifo A, Jo J, Price-Whelan A, McDonald ST, Brown LM, Sieben C, Dietrich LEP. 2021. *Pseudomonas aeruginosa* PA14 produces R-bodies, extendable protein polymers with roles in host colonization and virulence. Nat Commun 12:4613.

7. Fazli M, Harrison JJ, Gambino M, Givskov M, Tolker-Nielsen T. 2015. In-frame and unmarked gene deletions in *Burkholderia cenocepacia* via an allelic exchange system compatible with Gateway technology. Appl Environ Microbiol 81:3623-30.

8. Hoang TT, Karkhoff-Schweizer RR, Kutchma AJ, Schweizer HP. 1998. A broad-host-range Flp-FRT recombination system for site-specific excision of chromosomally-located DNA sequences: application for isolation of unmarked *Pseudomonas aeruginosa* mutants. Gene 212:77-86.
