# Supplementary material for: Systematic identification of molecular mediators of interspecies sensing in a community of two frequently coinfecting bacterial pathogens
Source: PLoS Biol. 2022 Jun 21;20(6):e3001679. doi: 10.1371/journal.pbio.3001679 (PMC9249247; doi:10.1371/journal.pbio.3001679)
Supplement: S3 Table — (PDF) [file pbio.3001679.s012.pdf]

S3 Table. Primers used in this study.

Primer	Sequence 5′→3′	Site^	Location*	Application
Pa020	atcccgacgggcccggtaccactagtcaggtaagcgcattccgc	SpeI	F P'PA14_73020	Promoter-reporter
Pa024	atcccgacgggcccggtaccactagtcagctggcgccaatcctc	SpeI	F P'PA14_11320	Promoter-reporter
Pa029	atcccgacgggcccggtaccactagtcgggaagacagggagaaatc	SpeI	F P'PA14_10290	Promoter-reporter
Pa031	atcccgacgggcccggtaccactagtgcgagctgcggatgatct	SpeI	F P'PA14_54520	Promoter-reporter
Pa045	atcccgacgggcccggtaccactagtgcgggaggaattggagat	SpeI	F P'PA14_63960	Promoter-reporter
Pa094[#]	ccgttcatagaaccctccatg	-	R mScarlet	Sanger sequencing
Pa110	atctccttcttaaatctagactcgaggggctgaacctgggaaat	XhoI	R P'PA14_73020	Promoter-reporter
Pa112	atctccttcttaaatctagactcgaggaacgggactccggcaag	XhoI	R P'PA14_11320	Promoter-reporter
Pa113	atctccttcttaaatctagactcgaggtccacatggtccttcgagt	XhoI	R P'PA14_10290	Promoter-reporter
Pa114	atctccttcttaaatctagactcgagggaagtcgacatgcagtggt	XhoI	R P'PA14_54520	Promoter-reporter
Pa121	atctccttcttaaatctagactcgagaccgaggacaagcgacac	XhoI	R P'PA14_63960	Promoter-reporter
Pa127	ggggacaagtttgtacaaaaaagcaggctcacgaagttcaccagggtcagt	*attB1*	Up PA14_63960	Generate mutant
Pa128	ggcggtagagggctcagtacatgggaaatcgcaccag	-	Up and down PA14_63960	Generate mutant
Pa129	ctggtgcgatttcccatgtactgagccctctaccgcc	-	Up and down PA14_63960	Generate mutant
Pa130	ggggaccactttgtacaagaaagctgggtaacaaatgtgcgccgacct	*attB2*	Down PA14_63960	Generate mutant
Pa131[#]	gaaatgcagcggatcgag	-	Up PA14_63960	Sanger sequencing
Pa132[#]	agtaccgcgcttttctgct	-	Down PA14_63960	Sanger sequencing
Pa133	ggggacaagtttgtacaaaaaagcaggctcagtgcgagctgagcgaact	*attB1*	Up PA14_63910	Generate mutant
Pa134	ggaggctcagcccttcttcttcagcaggtcgagcac	-	Up and down PA14_63910	Generate mutant
Pa135	gtgctcgacctgctgaagaagaagggctgagcctcc	-	Up and down PA14_63910	Generate mutant
Pa136	ggggaccactttgtacaagaaagctgggtagctgctctacagcatctcgac	*attB2*	Down PA14_63910	Generate mutant
Pa137[#]	ccggatactttccgagcag	-	Up PA14_63910	Sanger sequencing
Pa138[#]	acggcagtccatggaagat	-	Down PA14_63910	Sanger sequencing

Pa139	ggaggctcagcccttcttcatgggaaatcgcaccag	-	Down PA14_63910 and up PA14_63960	Generate mutant
Pa140	ctggtgcgatttcccatgaagaagggctgagcctcc	-	Up PA14_63960 and down PA14_63910	Generate mutant
Pa157	atcccgacgggcccggtaccactagtggaaatcaccttgctgcgg	SpeI	F P'PA14_33270	Promoter-reporter
Pa158	atctccttcttaaatctagactcgagctgccgacctccttgcg	XhoI	R P'PA14_33270	Promoter-reporter

* F = forward of ORF; R = reverse of ORF; up = upstream arm of gene; down = downstream arm of gene.

# Primers utilized for Sanger sequencing.

^ Site is underlined in primer sequence.
