# Supplementary material for: Systematic identification of molecular mediators of interspecies sensing in a community of two frequently coinfecting bacterial pathogens
Source: PLoS Biol. 2022 Jun 21;20(6):e3001679. doi: 10.1371/journal.pbio.3001679 (PMC9249247; doi:10.1371/journal.pbio.3001679)
Supplement: Attachment — Submitted filename: 20220317_Zarrella_Khare_Response_to_reviews.pdf [file pbio.3001679.s013.pdf]

We are grateful to the reviewers for their valuable suggestions and comments that we believe have enhanced our manuscript. We have responded to all of their concerns, performing additional experiments to address the possibility that the *P. aeruginosa* responses are due to a general starvation response, as well as to test the prevalence of the identified responses in *P. aeruginosa* clinical isolates. Additionally, we have clarified some methodological details, added labels and details to several figures and figure legends, and expanded on findings from previous studies. Our point-by-point responses to the comments are denoted below in bold type.

Thank you for submitting your manuscript "Systematic identification of molecular mediators of interspecies sensing in a two-species bacterial community" for consideration as a Research Article at PLOS Biology. Your manuscript has been evaluated by the PLOS Biology editors, an Academic Editor with relevant expertise, and by three independent reviewers. Please accept my apologies for the delay over the holiday period.

You'll see that while all three reviewers are impressed by the scale of your study, and reviewers #2 and #3 are overall broadly positive, reviewer #1 raises significant concerns about the possibility that you are merely observing a generic starvation response, and the limited connection to clinical situations. I discussed this discordance of opinion with the Academic Editor, who said that you should "address the critical comments of Reviewer #1 by performing a few additional experiments, particularly to rule out that what they see is a general starvation response. This should be relatively straightforward... In principle, even testing few clinical strains to confirm that they exhibit similar responses as PA14 (without redoing the screen) should not be that difficult." I hope these comments are useful in helping you to decide how to revise your manuscript.

In light of the reviews (below), we will not be able to accept the current version of the manuscript, but we would welcome re-submission of a much-revised version that takes into account the reviewers' comments. We cannot make any decision about publication until we have seen the revised manuscript and your response to the reviewers' comments. Your revised manuscript is also likely to be sent for further evaluation by the reviewers.

****
REVIEWERS' COMMENTS:

Reviewer #1:

In the manuscript Systematic identification of molecular mediators of interspecies sensing in a two-species bacterial community, Zarrela and Khare studied the metabolites secreted by S. aureus that influenced the metabolism of P. aeruginosa using a systems biology approach. This referee acknowledged the amount of work that is presented in this work. However, I have important reservations related to the study design and the experimental outcome of this work:

-The strain selection. Patients with cystic fibrosis usually develop long-term infections. S. aureus and P. aeruginosa are often found in the airways of these patients. Studies show higher prevalence of P. aeruginosa than S. aureus in older CF patients as it is known that P. aeruginosa replaces S. aureus during the course of the infection. More recently, it has been shown that that the two species are able to coexist in the lungs of CF patients. Because of this, the interspecies competition/coexistence of these two bacterial species is one of the most

studied case of multispecies community in microbiology. Therefore, it is important to study these interactions in an environmental niche the closely mimics the CF disease. The strain of P. aeruginosa used in this study is PA14, a classical isolate for laboratory studies which is not a CF strain. P. aeruginosa rapidly adapts to the host immune responses and antibiotic treatments of CF patients. This adaptation involves the accumulation of genetic mutations that alter genes expression and phenotypes of P. aeruginosa (Smith et al., 2006; Marvig et al., 2015a; Winstanley et al., 2016; La Rosa et al., 2019). Therefore, CF strains of P. aeruginosa differ from PA14, as they are specifically adapted to persist in a CF infection niche. In the case of the S. aureus strain, the authors did not use a clinical isolate but a genetically-modified S. aureus strain JE2. This strain JE2 or any other strain with a similar genetic background will not be found in any infection, as it results from genetic manipulation in a laboratory. The strain JE2 does not have the plasmids which confer to MRSA strains their multi-drug resistance profile as well as gene expression modifications. Overall, the use of these PA and SA strains does not represent the interaction niche of a CF microbial community.

We appreciate the concern that *P. aeruginosa* and *S. aureus* strains differ greatly during the course of long-lived infections in cystic fibrosis (CF). Therefore, we tested both the ability of *S. aureus* to induce responses, as well as the induction of *P. aeruginosa* responses, in clinical isolates of both species. In Fig. 7, we had showed that supernatants from four *S. aureus* CF clinical isolates were able to induce all four *P. aeruginosa* PA14 promoter-reporter strains, albeit to different degrees. Further, we had measured citrate and acetoin levels in each isolate supernatant and this correlated with the level of induction of the respective promoters.

We have now performed additional experiments to test *P. aeruginosa* clinical isolates. As shown in the new Fig. S6, we introduced each of the four promoter-reporter constructs into four *P. aeruginosa* CF clinical isolates from different patients (Table S14) and measured their induction. All the clinical isolates tested induced each promoter after exposure to *S. aureus* supernatant, except CF72 where the promoters of *pvdG* and *acoR* were not induced. These data have been described in the text (page 14 lines 349-353). Thus, these data demonstrate that the sensing and response pathways we have identified are commonly seen in *S. aureus* and *P. aeruginosa* strains which are found in CF infections.

-The methodology. The authors used cell-free exhausted supernatant from S. aureus cultures to supplement P. aeruginosa cultures. They authors claimed that, in 25% V/V supplemented P. aeruginosa cultures, this bacterium will respond to the presence of S. aureus secreted biomolecules. This is partially true. The most important effect associated with this medium supplementation will be a starvation/stress response that is consequence of 25% nutrient deprivation. Adding 25% of exhausted growth medium to the culture will affect growth of P. aeruginosa, irrespective on whether the exhausted medium was obtained from a S. aureus culture or any other species. Using this methodology, the three most important responses of P. aeruginosa will be the following: 1) a substantial starvation response that is consequence of a 25% nutrient deprivation., This will induce the genes related to the TCA cycle, to enable the bacterium to use alternative metabolic sources, such as citrate (see the following point). The starvation response also includes low availability of metal ions, which induces the secretion of metal chelators. 2) A stress response that is consequence of a pH variation, which includes the expression of genes involved in the ROS response.

To avoid these effects, the cultures supplementation studies are usually carried out using

exhausted medium corrected in pH and nutrient availability, in such they are comparable to that of other regular growth media.

- The bacterial response. The authors claimed that the strongest P. aeruginosa response to S. aureus cell-free supernatant was represented by four pathways: Zn-deprivation, Fe-deprivation, TCA uptake, and acetoin catabolism. This is however, a classical starvation response which likely is a consequence of the 25% nutrient deprivation of supplemented cultures. in other words, the authors will obtain a comparable response by complementing PA cultures with spent media from any other cultures or even with low-pH water supplemented with alternative metabolic sources such as citrate or acetate. This is because, during exponential growth in laboratory cultures, carbohydrates are quite available and P. aeruginosa uses respiration to grow (via TCA cycle) until the concentration of carbohydrates decreases. Only then do the bacteria rely on other metabolic sources, such as citrate or acetate, to feed the TCA to obtain energy. This involves an induction of the TCA cycle to maintain an effective growth, as citrate or acetate are not energetically as efficient as are the carbohydrates. This occurs indeed during starvation conditions. The same applies to the deprivation of metal ions, which are essential for many enzymatic reactions. A 25% nutrient deprivation in the supplemented cultures causes a defect metal ions availability; bacteria reacted to this effect by inducing the production of metal chelators.

Overall, I think the system biology approach presented in this work is quite interesting but the experimental outcome does not allow to obtain important information to understand the interaction/coexistence of P. aeruginosa and S. aureus in the lung of CF patients.

The reviewer raises an important possibility that the *P. aeruginosa* responses we are seeing may be due to a general starvation response. We have performed additional experiments to test this, and believe several lines of evidence suggest against this possibility:

a) **pH: *S. aureus* spent cell-free supernatant has a pH similar to the medium, and when this supernatant is added at 25% (v/v) to medium (which is the concentration used for all assays), it does not affect the pH compared to medium control (this data is now shown in <u>Fig. S2A</u>; text on page 7 lines 165-168)**
b) **Nutrient availability: We performed a new experiment to test whether reduced nutrient availability in the 25% (v/v) *S. aureus* supernatant is sufficient to induce the promoter reporters. First, addition of 25% (v/v) of just the salts from the medium did not induce any of the promoter-reporters (new <u>Fig. S2C</u>), indicating that nutrient deprivation did not underlie the responses we were seeing. Second, we lyophilized *S. aureus* supernatant, resuspended it in either water or complete media as a control, added it at 25% (v/v) to the medium and tested its ability to induce the promoter-reporters. All four promoter-reporters were still induced (<u>Fig. S2C; pages 7-8 lines 168-177</u>). Induction of the *pvdG* promoter indicating iron starvation was lower when lyophilized *S. aureus* exoproducts were resuspended in media, likely due to the increased iron availability. Induction of *acoR* was lower in response to lyophilized supernatant, regardless of resuspension in media or water, suggesting that lyophilization may reduce active metabolite concentration.**
c) **Species-specificity: In <u>Fig. S7</u>, supernatants from 8 other species were added to each promoter reporter strain. Induction of the promoter-reporters was not universal among the species, and each promoter was induced by different combinations of 2-4 species, indicating that the response is not a general starvation response.**

d) ***P. aeruginosa* growth: The RNA for the RNA-seq experiments was collected within 2 hours, and the slopes of reporter expression were calculated within the first 5 hours. During this time, *P. aeruginosa* does not show a growth difference when grown in 25% (v/v) *S. aureus* supernatant vs. medium (data now shown in <u>Fig. S2B</u>), suggesting that *P. aeruginosa* is unlikely to be facing starvation conditions during this time. This is likely because the culture medium used (M63) is a rich defined medium containing glucose as well as amino acids that can serve as carbon sources, and *P. aeruginosa* cultures were grown in fresh medium to an OD_{600} of only 0.5 (early exponential phase) before the addition of 25% (v/v) of media or supernatant.**

Reviewer #2:

In this paper by Zarella and Khare, the authors dissect multiple stimuli that influence P. aeruginosa that are present in S. aureus supernatants. The paper summarizes a lot of work and is pitched as a system for the discovery of metabolites mediating interaction. The major factors in S. aureus supernatants that affect the P. aeruginosa transcriptome include the zincophore staphylopine, iron binding factors, citrate and acetoin. The work melds the use of transcriptomics and fractionation to genetic screens of a mutant S. aureus library to identify mutants that no longer trigger these responses. I think that the approach of combined transcriptomics with genetic screens has been well-used, a fact that may be underrecognized in the introduction/set-up of the paper.

The major contribution of this paper is in the final figures in which the authors look at the additive effects of the different S. aureus stimuli on the overall P. aeruginosa response which is an exciting next step in microbe-microbe interactions research and show that these responses are observed even if other clinical strains are used. I am really impressed with Figures 7 (clinical isolates) and in particular, Fig. 8. Line 105-8-the approach aims to quantify the fraction of the transcriptional response that can be explained by the different small molecule stimuli. In the end, about ~50% of the response (97 genes of 184 genes) to supernatant could be explained by four different stimuli. This nicely highlights what was achieved and what remains to be explained.

We thank the reviewer for appreciating the approach and findings in the paper and have addressed each of their specific comments below.

Specific comments:

1. Table S5. It would be helpful if the cnt mutants that were identified in the screen in Table S5 were identified. (I was not able to determine which genes were identified in the screen using publicly available databases.) It is not clear that the cnt mutants were the top candidates in the screen or if they were pulled from the collection based on their known function and analyzed in a targeted fashion in Fig. 2. The top hits in the table did not seem to be involved in StP biosynthesis.

The genes encoding CntE and CntKLM are now denoted in a separate column in <u>Table S5.</u> Since multiple *cnt* mutants were hits from the screen (including 2 of the top 6 hits after excluding mutants with growth defects), we decided to investigate this pathway further. Individual colonies of the transposon mutants were confirmed for the respective

transposon insertions and then validated for reduced induction of the PA14_11320 promoter (Fig. 3B). We have now clarified this in the text (Page 8 lines 185-191).

2. Please include the specifics for the z-score transformations.

The information to calculate the z-scores is now included in the Materials and Methods section under the Plate reporter assay heading (page 25 lines 635-638), as follows: "To calculate the z-score, for each individual experiment (96-well plate) the mean slope and standard deviation were determined. Each sample replicate z-score is the number of standard deviations away from the mean, calculated using the formula $z = (x-\mu)/\sigma$, where x is the sample slope, μ is the mean slope, and σ the standard deviation."

3. The data in Fig. 5ABC and Fig. 6ACD seems peripheral and in the end, particularly for Fig. 6, the data didn't strongly support a correlation between levels. I suggest moving to the supplement (despite the fact that they represent a lot of work). The inclusion of these data make this data-rich paper harder to follow.

We appreciate the reviewer's concern that Fig. 5ABC and 6ACD make the paper more difficult to follow. However, we believe that in both cases, the figures exemplify how promoter-reporter screens, in conjunction with either GO enrichment (specific metabolic pathways in Fig. 5B) or gene annotations (citrate metabolism genes highlighted in 5A, butanoate pathway genes highlighted in 6A, and metabolic pathways in 5C and 6C) can help to identify the sensed molecules. While the correlation between acetoin levels and promoter induction (Fig. 6D) is lower than that for citrate, it is highly significant (Pearson's *r* = 0.2157, *p* < 0.0001). Further, in both cases, the data demonstrate the additional utility of using our approach to determine on a genome-wide scale the genes that are involved in making the sensed exoproduct.

We therefore believe that including these data in the main figures would aid readers in better understanding our approach and results, and have thus retained them in the main figures. We realized we did not originally highlight this added advantage of our strategy in delineating on a genome-scale the pathways that affect the production of the *S. aureus* sensed molecules in the main text, and have now included it in the abstract as well (Page 2, lines 36-38).

4. Please include more specifics on the z-score calculations.

This has been added as described above for comment #2.

5. Some references for these molecules in other P. aeruginosa interactions:

a) Please include mention of Mould et al. as evidence for extracellular citrate response inducing a P.a. response and the enhancement of this interaction with iron siderophores.
b) More clear reference to work by Skaar et al. on the topic of zincophores in P.a.-Staph interactions.
c) Recognize published interactions with S.a. such as P.a. production of HQNO and siderophores in co-culture not mentioned (or not mentioned clearly) by O'Toole and colleagues. Citrate and HAQs are particularly important given that they identify metal scavenging as one of the signals induced by Staph sup.

We have now expanded on the above references as follows:

Mould, et al. (92) is referenced in the Discussion (page 20 lines 495-496): "Higher concentrations of citrate affect biofilm formation in *P. aeruginosa* (60) and extracellular citrate can stimulate quorum sensing in strains deficient for the LasR regulator (92)."

Wakeman, et al. (last author Skaar) (57) is referenced in the Results (page 9 lines 225-226): "Higher levels of zinc availability are associated with increased *P. aeruginosa* biofilm formation and increased antagonism of *S. aureus* (57-59)" and in the Discussion page 18 lines 443-448): "Interactions between *P. aeruginosa* and *S. aureus* in the context of zinc deprivation have been studied before in the presence of a neutrophil-derived metallophore, calprotectin, which binds zinc, manganese, nickel, and iron. Through metal chelation, calprotectin decreases anti-staphylococcal antimicrobial production and inhibits extracellular protease-mediated lysis of *S. aureus* thereby promoting coexistence between *P. aeruginosa* and *S. aureus* (57,58)."

Filkins, et al. (last author O'Toole) (34) has now been cited again twice in the Discussion (pages 18-19 lines 461-463): "In a previous study, more *S. aureus* was recovered from co-culture with a *P. aeruginosa* pyoverdine-deficient strain than WT, suggesting that pyoverdine is produced in the presence of *S. aureus* exoproducts and has an antagonistic effect on *S. aureus* (34)." and (page 20 lines 489-492): "Previously, it was reported that in the presence of *P. aeruginosa* exoproducts *S. aureus* secretes lactate which is consumed by *P. aeruginosa* (34). However, our *S. aureus* supernatant was obtained from monocultures, and likely lacks production of this metabolite."

6. Confirm that all figure legends specify which promoter fusion was used-in at least two places it was necessary to look in the results for this info.

Information about the promoter-reporters was added to the figure legends (underlined) in Fig. 3: "(B-F) RFU of mScarlet normalized to OD_{600} over time from 1.5 to 5 h after exposure to media control or *S. aureus* WT or *cnt* supernatant and/or addition of the indicated concentrations of StP, Zn, or TPEN in the indicated *P. aeruginosa* WT or mutant promoter-reporter strains <u>(promoters of PA14_11320, *cntO*, or *dksA2*)</u>." and Fig. 4: "(C) RFU of <u>P'*pvdG*-mScarlet</u> normalized to OD_{600} over time from 1 to 4 h after exposure to media control, 1X whole *S. aureus* WT supernatant, water, or 10X fractions with or without proteinase K treatment (mixed 1:1 with medium)."
Additionally, based on suggestions from both Reviewers 2 and 3, several labels were added to the figures (described in the responses to Reviewer 3).

7. Lines 187-188 "indicating that similar concentrations are likely present in staphylococcal supernatant." This is a strong statement. It would be more appropriate to say that this concentration is sufficient to induce to levels noted with supernatant induction. (This is especially in light of data showing that stp mutant supernatant still induced higher levels of the zinc responsive promoter than media control to suggest the presence of an additional factor.)

The text has been changed as suggested, and now reads (page 9 lines 208-211): "…indicating that this concentration is sufficient to induce the promoter to a level similar to that of WT supernatant."

8. Line 194. The data on P. aeruginosa cnt mutants is not relevant to the response to Staph supernatants. These data should be moved to the supplement or removed.

We are also interested in how the molecules we identify affect interspecies responses and interactions. Since *P. aeruginosa* produces a very similar molecule, it was important to determine if these molecules synergize, or act in competition, for metals. The data in Fig. 3F suggest that the pseudopaline pathway aids in protection from staphylopine-induced zinc-starvation responses, providing new insight into *S. aureus* – *P. aeruginosa* interactions. We have now expanded on our discussion of these results to clarify this (page 9 lines 219-222): "Upon supernatant addition, induction of mScarlet reporter expression was increased in the *P. aeruginosa* Δ*cntO* and Δ*cnt* mutants compared to the WT (Fig. 3F), demonstrating that the presence of PsP partially protects from StP-induced zinc starvation, indicating that the two metallophores compete for zinc."

9. Throughout, for the phenotype assays, it would be useful to know where there are differences in growth rather than only showing the OD normalized data. The data in Fig. 2 show a delay rather than a lack of induction for zinc and iron responses which makes for a very strong response in the 1-4 hour window. Based on the pattern for data in Fig. 2A for 11320, it seems that the overnight cultures were limited for zinc. Please note when differences in growth are observed.

Growth data for *P. aeruginosa* in the plate reader assay exposed to 25% (v/v) supernatant or media control is now shown in Fig. S2B (page 7 lines 165-168). There appear to be no differences in growth during the first ~6 hours which is when we measure the slopes for the promoter-reporters, and thus growth differences are unlikely to affect the normalization calculations. In addition, the experiments are done with *P. aeruginosa* sub-cultured from an overnight culture, grown to OD_{600} of 0.5 in fresh medium, and then added to the plate 75% to 25% (v/v) *S. aureus* supernatant or media control. The media contains 0.8 μM ferric citrate, and trace amounts of zinc, and the exponential phase cultures used in the assays appear to be replete for these metals, as there is no difference in growth between the medium control and *S. aureus* supernatant conditions in the first 6 hours.

Reviewer #3:

Summary
The authors have developed an elegant approach to identify both the interspecies signals and the mechanisms of sensing in a two-member bacterial community by combining transcriptomics, genetic screens, and proteomics approaches. The authors utilized the well-characterized pair P. aeruginosa and S. aureus, which allowed for the identification of previously published pathways, helping to validate the utility of the approach, but also previously unidentified interactions. The authors also followed up briefly on the four major pathways identified (zinc and iron deprivation, TCA intermediate uptake, and acetoin metabolism) with genetic and phenotypic studies yielding deeper insight into interactions between these important pathogens. The paper is extremely well-written and the figures are beautifully designed. I have only a few very minor suggestions below.

We are thankful for the positive comments from the reviewer about our study design, results and presentation, and have accepted all their suggestions, as detailed below.

1. The authors remark that this approach can exhaustively reveal the molecules that lead to

response of a foreign species. While I am impressed with the authors' multi-technique approach, I wonder one validates that it is exhaustive?

The use of representative promoter-reporters from all coordinately regulated pathways could potentially identify all the molecules that lead to the response in a foreign species, which is what we wished to convey. However, we appreciate the reviewer's point that this may be practically very difficult to accomplish and validate. We have therefore changed the wording to now read (page 6 lines 124-127): "This two-pronged unbiased approach has the potential to comprehensively reveal the molecules that lead to complex responses in a foreign species, irrespective of which pathways and mechanisms constitute the response."

2. The clarity of Figure 3 could be improved with more detailed labeling on the Figure and in the text description. Some suggestions:
* More specifically label "+ sup". This is WT S. aureus sup?
* In the text, more indicating which organism the genes are deleted from more often, would help the reader keep track.
* In 3C, the blue control WT PA14 with StP in the absence of S. aureus supernatant? Does this result in a significant increase in P'11320 and suggest it is sufficient?
* For figures where only one promoter is examined, it may help to indicated that on the Y-axis (P'11320 for 3C-F, I think).

In Fig. 3C, adding 40 or 80 µM staphylopine to media did increase the slope means, however these data were not significantly different from the media control. It is possible that *S. aureus* has other zinc-binding factors, which is why the addition of these amounts to only the *S. aureus cntM* mutant supernatant restored induction levels.

Fig. 3 has been updated with the following changes: "+ Sup." changed to "+ WT *S. aureus* Sup.", "*cntM*::tn" changed to "+ *cntM*::tn Sup."; if the promoters are not already indicated on the *x* label, this has been added to the *y*-axis. In addition, the promoters are now indicated on the *y*-axis in Fig. 4C and in the titles of the following panels: Fig. 6B, S4B, S5A.

In the Results section, several additions were made to clarify the species of the mutant being referenced (page 9, lines 208-210 and 219-221): (underlined) "The addition of increasing amounts of StP to the *S. aureus* *cntM*::tn mutant supernatant restored P'PA14_11320 induction to WT levels at concentrations greater than 40 µM …" and "Upon supernatant addition, induction of mScarlet reporter expression was increased in the *P. aeruginosa* Δ*cntO* and Δ*cnt* mutants compared to the WT…"

3. Model: Unless grown under swarming conditions, P. aeruginosa typically produce a single polar flagellum.

The model in Fig. 8 has been updated with a new *P. aeruginosa* with one polar flagellum.

In compliance with data protection regulations, you may request that we remove your personal registration details at any time. (Use the following URL: https://www.editorialmanager.com/pbiology/login.asp?a=r). Please contact the publication office if you have any questions.
