# Supplementary material for: Systematic identification of molecular mediators of interspecies sensing in a community of two frequently coinfecting bacterial pathogens
Source: PLoS Biol. 2022 Jun 21;20(6):e3001679. doi: 10.1371/journal.pbio.3001679 (PMC9249247; doi:10.1371/journal.pbio.3001679)
Supplement: Attachment — Submitted filename: 20220516_response.pdf [file pbio.3001679.s014.pdf]

We would like to thank the reviewer and editors for their helpful suggestions. Our point-by-point responses to the comments are denoted below in bold type.

a) Please change your title to "Systematic identification of molecular mediators of interspecies sensing in communities of two frequently co-infecting bacterial pathogens"

The title has been changed to "Systematic identification of molecular mediators of interspecies sensing in a community of two frequently co-infecting bacterial pathogens"

b) You will see that reviewer #1 continues to raise some concerns related to those mentioned in the first round. The Academic Editor kindly discussed these issues with the reviewer, and has given me specific instructions as to how you should address these: "I would ask the authors to address the first two points that reviewer #1 raises, namely

1) to explain the rationale for selection of the clinical isolates that they now tested

The isolates tested were drawn from those sent from the Cystic Fibrosis Foundation Isolate Core. The main rationale for selecting them was to select isolates from multiple patients. For *S. aureus*, four isolates were picked from three different patients, with two from the same patient - one that was co-isolated with *P. aeruginosa* and one mono-isolated. For *P. aeruginosa*, four isolates were picked from four different patients.

These, and additional details about the isolates have been added to the text now:
- The patient identifiers and patient ages at which the isolates were collected have been added to S14 Table.
- The strain selection has been added to the Materials and Methods section (page 22 lines 704-706): "*S. aureus* and *P. aeruginosa* clinical isolates from the Cystic Fibrosis Foundation Isolate Core were selected from different patients, with two *S. aureus* isolates from the same patient, one of which was mono-isolated and the other co-isolated with *P. aeruginosa.*"
- In the Results (Page 18-19, lines 455-458 and 467-470):
"To test how widespread the production of these molecules is, we surveyed supernatants from four clinical isolates of *S. aureus* that were mono-isolated (CF049 strain) or co-isolated with *P. aeruginosa* (CF061, CF085, and CF089 strains) from three different cystic fibrosis patients."
"Additionally, to test how widespread the identified *P. aeruginosa* responses are, four *P. aeruginosa* clinical isolates (CF17, CF33, CF72, CF104) that were co-isolated with *S. aureus* from four different cystic fibrosis patients were transformed with each promoter-reporter construct."

2) to better discuss the variability of responses or their induction among clinical isolates, since not all of them behave the same way. As of now, their statement in the Abstract 'Cystic fibrosis clinical isolates of both S. aureus and P. aeruginosa also showed induction or responses, respectively, which suggests that these interactions are widespread among pathogenic strains' does not reflect this variability among isolates."

We have now clarified the variability of the induction and sensing phenotypes among clinical isolates:

(page 2 lines 41-44): "Cystic fibrosis clinical isolates of both *S. aureus* and *P. aeruginosa* also showed varying degrees of induction or responses, respectively, which suggests that these interactions are widespread among pathogenic strains."

(pages 4-5 lines 99-101): "Several clinical isolates of *S. aureus* and *P. aeruginosa* show induction and responses respectively of varying intensity, and other bacterial species can also induce some of the same pathways in *P. aeruginosa*, indicating the generality of these phenomena."

(page 23, lines 586-589): "Further, production and sensing of the sensed molecules was conserved in most clinical isolates of *S. aureus* and *P. aeruginosa*, respectively, suggesting that these pathways and interactions are common between these two species."

(page 27, lines 677-680): "While our screen was carried out in a single strain of each species, we validated that *S. aureus* clinical isolates from cystic fibrosis patients, including strains that were co-isolated with *P. aeruginosa*, also produced molecules that induced the sensory pathways, albeit at varying levels, and that most *P. aeruginosa* clinical isolates also induced the response pathways."

c) Please address my Data Policy requests below; specifically, we need you to supply the numerical values underlying Figs 1CDE, 2AB, 3BCDEFG, 4ABCDE, 5ABDE, 6ABDE, 7ABCD, 8ABCD, S1, S2ABC, S3ABCD, S4ABC, S5AB, S6, S7, S8ABCD. Please supply these values, either as a supplementary data file or in a recommended repository. I see that you already present a large amount of data in your "Supplemental_File_2," but it's currently unclear how this relates to the Figs. Please also cite the location of the data clearly in each relevant main and supplementary Fig legend, e.g. "The data underlying this Figure can be found in S1 Data."

These values have been supplied in various supplementary tables including S16 Table, which is a separate file. Statements have been added to each main and supplementary figure legend regarding the availability of the underlying data.
